# Aluminum-Free Borosilicate Glass Functionalized Hydrogels for Enhanced Dental Tissue Regeneration

**DOI:** 10.3390/ma17235862

**Published:** 2024-11-29

**Authors:** Nina Attik, Inès Basri, Jérôme Sohier, Rémy Gauthier, Cyril Villat, Christelle Goutaudier

**Affiliations:** 1 Laboratoire des Multimatériaux et Interfaces UMR CNRS 5615, Universite Claude Bernard Lyon 1, 6 rue Victor Grignard, 69622 Villeurbanne, Francecyril.villat@univ-lyon1.fr (C.V.); christelle.goutaudier@univ-lyon1.fr (C.G.); 2 Faculté d’Odontologie de Lyon, 11 rue Guillaume Paradin, 69008 Lyon, France; 3 Laboratoire de Biologie Tissulaire et Ingénierie Thérapeutique, Universite Claude Bernard Lyon 1, UMR 5305 CNRS, 69367 Lyon, Cedex 7, France; jerome.sohier@cnrs.fr; 4 MATEIS, CNRS, INSA Lyon, UCBL, University Lyon, UMR5510, 20 Avenue Albert Einstein, 69621 Villeurbanne, France; remy.gauthier@cnrs.fr; 5 Hospices Civils de Lyon, Service d’Odontologie, 69007 Lyon, France

**Keywords:** borosilicate bioactive glass, cytocompatibility, dental tissue regeneration, functionalized hydrogels, porosity, remineralization, shapeable scaffolds

## Abstract

Hydrogels are promising scaffolds for tissue regeneration, and borosilicate glass particles have demonstrated potential in enhancing the biological behaviour of dental pulp cells. However, the specific morphological characteristics of dental lesions and the diverse requirements of dental tissues require biocompatible, bioactive, and shapeable scaffolds. This study aimed to evaluate the in vitro biological behaviour of human gingival fibroblasts (HGFs) in contact with an experimental aluminum-free borosilicate glass-functionalized hydrogel. Two types of experimental borosilicate glass particles were utilized, with Biodentine^®^ particles serving as a reference material. The hydrogel, based on poly(L-lysine) dendrimers (DGL) with or without borosilicate particles, was analyzed using micro-computed tomography (µCT) and scanning electron microscopy (SEM) coupled with energy-dispersive X-ray spectroscopy (EDX). Cytocompatibility was assessed using Live/Dead™ staining, and cell colonization was evaluated via confocal imaging. Additionally, Alizarin red staining was performed to assess mineralization potential after 7 and 14 days. Results indicated that the incorporation of borosilicate particles did not alter hydrogel porosity, while EDX confirmed particle presence on the hydrogel surfaces. Furthermore, the borosilicate-functionalized hydrogels significantly enhanced cell proliferation, colonization, and the content of calcium deposits. These findings highlight the potential of these hydrogels for future clinical applications in dental tissue regeneration, pending further development.

## 1. Introduction

Bioactive glasses have been extensively researched over five decades for their regenerative therapy potential [1]. The 45S5 glass, pioneered by Professor Hench, is notable for its ability to promote osteogenesis and integrate with bone tissue [2]. However, to enhance its clinical applications, numerous studies have been conducted to modify its biological or mechanical characteristics. This includes improving its dissolution rate, apatite-forming ability, antibacterial and anti-inflammatory activities, and stimulating angiogenesis [2,3]. These improvements have been achieved by incorporating elements, such as silver, fluoride, calcium, strontium, magnesium, zinc, copper, boron, silicon, and phosphate [2,4,5,6]. Moreover, bio-glasses based on various oxides, such as borosilicate glasses, have shown promising bioactive properties for bone repair and drug delivery systems [7]. These glasses degrade more rapidly than their silicate counterparts and are also known for exhibiting phase separation [8]. In our previous study, we examined a series of novel borosilicate glasses with enhanced phase separation and ion release profiles [9]. Westhauser et al. [10] demonstrated that a new borosilicate-based bioactive glass exhibited superior osteogenic performance both in vitro and in vivo compared to the well-known 45S5-BG. This finding aligns with our recent data, which showed an enhancement of dental pulp cells’ metabolic activity, spreading, and acellular bioactivity via apatite forming ability in vitro when in contact with borosilicate bioactive glass without alumina [11]. Our recent research has focused on developing aluminum-free borosilicate glasses to avoid potential neurotoxic effects, such as neurobehavioral damage from oxidative stress and impacts on mitochondrial cell functions [12].

On the other hand, hydrogels have gained significant prominence in the field of dental tissue regeneration, particularly for applications aimed at regenerating tissues, such as periodontal ligament [13] dentin, and pulp [13,14,15]. Their appeal lies in their biocompatibility, biodegradability, ability to release drugs in a controlled manner, unique swelling characteristics, and ease of fabrication [5,16]. The properties of hydrogels are highly influenced by their chemical or physical crosslinking mechanisms [16]. These mechanisms not only determine the structural integrity and mechanical properties of the hydrogel but also regulate its capacity to encapsulate and release bioactive agents crucial for promoting dental tissue regeneration [17].

In their review, Huang et al. highlighted that bioactive glass nanoparticles are widely used in biomedicine. However, they also pointed out some drawbacks, such as burst release and poor biological adhesion, indicating the need to combine these particles with a scaffold to overcome these limitations [18]. In this respect, researchers have explored technologies to deliver bio-glasses over an extended period by incorporating them into scaffolds, such as hydrogels, using 3D printing techniques [4]. Hydrogels can serve as bioactive-particle-delivering vectors, improving controlled release and preserving the bioactive properties of bioactive materials, such as the incorporated borosilicate particles [19]. Therefore, the application of these shapeable and borosilicate-functionalized hydrogels could effectively conform to the morphological features of dental lesions and accommodate the varying regeneration times of different dental tissues [20,21].

To develop a suitable carrier for experimental bioactive glasses, one of the most critical properties is flexibility to ensure effective incorporation within the pulp or targeted dental tissue [22]. Additionally, a porous structure is beneficial for enhanced biological performance, in terms of cellular infiltration, as well as vascularization and neurogenesis [23]. In this context, a hydrogel was synthesized with the following formulation: poly(L-lysine) dendrimers (DGL) and N-hydroxysuccinimide (NHS) bi-functionalized polyethylene glycol (PEG). This porous hydrogel formulation, previously developed, demonstrates a rapid and tunable crosslinking reaction, while inherently promoting cell interaction through polycationic charges provided by DGL, facilitating cellular adhesion and proliferation [24,25]. The hydrogel formulation was optimized for injectability and precise conformation to dental tissue defects.

Hence, the current study aimed to develop a borosilicate glass-functionalized DGL hydrogel for dental tissue regeneration. Three hypotheses were suggested:

1. The incorporation of borosilicate particles would influence the porosity of the DGL hydrogel.
2. The DGL hydrogel would facilitate the release of ions from the integrated borosilicate particles.
3. Borosilicate particles would enhance the behavior of human gingival fibroblasts in terms of cell proliferation, colonization, and mineralization potential.

## 2. Material and Methods

### 2.1. Hydrogel Elaboration and Particles Incorporation

#### 2.1.1. Used Particles

The particles used for this study were previously synthesized and characterized [26]. The phase-separated borosilicate glasses with the general quaternary formulation SiO_2_-K_2_O-B_2_O_3_-CaO were prepared using the melt-quenching technique. Several glass compositions have been synthesized by melting the raw materials at 1200–1300 °C in a quartz crucible for 4 h in an electrically heated furnace [9]. In this study, two formulation samples, without aluminum, are selected for the current experiments: PSBS8 with a high calcium content and PSBS3 with a higher boron content, with the silica and potassium oxide composition being constant. Biodentine^®^ (Septodont, Saint-Maur-des-Fossés, France) was used as a positive control. Biodentine^®^ is a hydraulic cement developed as a dentine replacement material and specifically targeted for vital pulp therapy, released in 2010. It has garnered significant interest in the dental and scientific communities [26,27]. The composition of the three tested is summarized in Table 1.

#### 2.1.2. Hydrogel Preparation and Functionalization

The formulation of the DGL/PEG polymer used in this study has been previously developed and adapted for the intended application [25]. In summary, the porous DGL/PEG hydrogel was prepared by mixing DGL (molecular weight of 22,000 g/mol, Colcom, Clapiers, France) and potassium carbonate (Sigma Aldrich, Saint-Quentin-Fallavier, France) at the desired concentration in PBS (Euromedex) in one conical tube (mix 1) to achieve a final volume of 200 μL. Pluronic^®^F-68 (Sigma Aldrich), glacial acetic acid, (Sigma Aldrich, Saint-Quentin-Fallavier, France), and PEG-NHS (PEG-NHS, 2000 g/mol, Sigma Aldrich, Saint-Quentin-Fallavier, France) in DMSO (Sigma Aldrich, Saint-Quentin-Fallavier, France) at the desired concentration were mixed in another conical tube (mix 2) to also obtain a final volume of 200 μL. Then, 2 mg of bio-glass particles (with a particle size of less than 100 µm) were incorporated into mix 1 and homogenized to prevent aggregation. Both mixes were thoroughly homogenized and placed in a 37 °C water bath. Subsequently, the mixes were transferred into a dual-chamber syringe at a 1:1 ratio and the final volume of 400 μL was injected into a 2.0 mL conical tube through a static mixing nozzle. After crosslinking, the hydrogel was immersed in 1 mL of ultrapure water. The porous hydrogels were then removed from the tubes prior to manual sectioning into 2 mm-high cylinder samples. This porous material was prepared at a final (50:75) DGL–PEG mass ratio. The reaction equation of the elaborated DGL/PEG hydrogel is shown below.

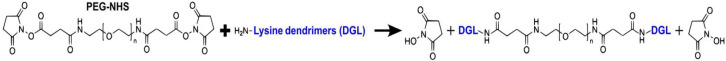



Where PEG refers to polyethylene glycol, NHS to N-hydroxysuccinimide, and DGL to poly(L-lysine) dendrimers

#### 2.1.3. SEM and EDX Analysis

Hydrogel samples surfaces analysis and the presence of particles within the DGL/PEG hydrogels were performed using a scanning electron microscope (SEM, FEI-Zeiss MERLI, Oberkochen, Baden-Württemberg, Germany) with an acceleration voltage of 10 kV. The EDX mode was used to investigate the surface composition of the tested functionalized hydrogels materials after sample coating using 10 nm of copper. Before SEM/EDX analysis, the tested hydrogels were softly dried by immersing them in aqueous solutions of increasing ethanol concentration to remove water from the samples.

#### 2.1.4. pH Measurement and Calcium Release Quantification by ICP-OES

Eight disks of each hydrogel were prepared as described above (8–10 mm diameter, with 2 mm thickness). Two discs of each material were placed in four test tubes with 6 mL of distilled water, and the pH was measured directly at 0 h, 7 h, 12 h, 24 h, 72 h, 168 h (7 days), 336 h (14 days), and 510 h (21 days) using a HI 9125 pH/ORP Meter (Sigma Aldrich, Saint-Quentin-Fallavier, France). After each pH measurement, the solution was recovered to perform the calcium release assay quantification by inductively coupled plasma–atomic emission spectroscopy (ICP, Vista MPX CCD Simultaneous ICP-OES, Varian Inc., Palo Alto, CA, USA).

#### 2.1.5. Micro-Computed Tomography (µCT) Analysis

The micro-computed tomography (μCT) analysis was conducted to obtain the pore size of each hydrogel as well as the volumetric porosity fraction of the tested samples. A high-resolution 3D image of the particle-functionalized hydrogels was obtained using an acceleration voltage of 60 kV up to 8000 × 8000 pixels, with 900 projections, 12 images per second, and a flat panel detector. The hydrogels samples were fixed (n = 3) using a 3.7% formaldehyde solution 15 min prior to μCT analysis (GE Phoenix V|tome|X, Munich, Germany).

#### 2.1.6. Thermal Behaviour Using TGA and DSC 

The decomposition profile of hydrogels DGL/PEG containing particles was performed using a dynamic mechanical analyzer (thermogravimetric Analysis (TGA)/differential scanning calorimetry (DSC) 3 apparatus by Mettler Toledo, Leipzig, Germany). Dried hydrogel quantities ranging from 0.5 to 5 mg were wrapped and placed in a platinum pan. All samples were heated from −120 °C to 25 °C and from 30 °C to 900 °C at a rate of 10 °C/min under nitrogen (N_2_), which served as both a protective and purging gas. The TGA/DSC data were plotted as temperature versus weight loss (%) and heat flow (mW/mg) to confirm the thermal behaviors of the hydrogels. Melting calibrations were performed using indium and mercury standards. The heat flux error was less than 1%, and the temperature error was ±0.2 K.

### 2.2. Cytocompatibility Assessment

#### 2.2.1. Cell Culture

The cytocompatibility assessment was achieved on human gingival fibroblasts (hGF). hGF cells were isolated from healthy gingival tissue biopsies of patients during routine orthodontic extractions. The collection of human dental tissue was conducted in compliance with French legislation and approval from the institutional ethics committee (Art R1211 CSP). The obtained cells were cultured in Dulbecco’s modified Eagle’s medium (DMEM) supplemented with 10% fetal bovine serum, 2% penicillin/streptomycin, and 1% amphotericin B. The cultures were maintained at 37 °C in a humidified atmosphere with 5% CO_2_. Once the cells reached confluence, they were trypsinized and resuspended in fresh culture medium. All culture reagents were purchased from Thermo Fisher Scientific (Villebon-sur-Yvette, France). The cells were then centrifuged at 1200 rpm for 5 min and counted using a Scepter handheld automated cell counter (Millipore, Billerica, MA, USA). After the removal of trypsin, the remaining cell pellets were resuspended in fresh medium for subsequent experiments and incubated overnight at 37 °C in a humid atmosphere of 5% CO_2_ in air. In total, 1 mL of cell suspension at a concentration of 10^4^ cells/mL was seeded in 24-well plates (130,186, Thermo Fisher Scientific, Villebon-sur-Yvette, France) in contact with hydrogel samples in the form of discs with a thickness of 2 mm and a diameter of 8 mm.

#### 2.2.2. LIVE/DEAD^TM^ Staining

The Live/Dead™ assay (L3224, ThermoFisher Scientific, Villebon-sur-Yvette, France) was conducted on cells directly interfacing with the tested hydrogels to evaluate potential cytotoxic effects, monitoring membrane integrity at 24 h. Calcein AM staining identified viable cells, while ethidium homodimer (EthD-1), a cell-impermeable dye for dead and dying cells, labeled damaged cells. A working solution comprising 2 µM calcein AM and 4 µM EthD-1 was applied to the cellularized samples (cells in contact with hydrogels). Microscopic analysis was conducted 10 min after staining using an epifluorescence microscope (EVOS™ M5000 Imaging System, Villebon-sur-Yvette, France). Excitation/emission wavelengths for calcein AM and EthD-1 were 495/515 nm and 525/635 nm, respectively.

#### 2.2.3. Cell Morphology and Spreading by Confocal Laser Scanning Microscopy (CLSM)

Fluorescence staining was conducted to observe the formation and organization of stress fibers and morphological changes. Cells were seeded in direct contact with the tested hydrogels, followed by incubation at 37 °C for 24 h. Both the cell control and cellularized hydrogels were harvested and washed three times with PBS. Subsequently, the cells were fixed by incubating them in 3.7% formaldehyde in PBS for 30 min, followed by several washing steps. Cell permeabilization was achieved with 1% Triton X-100 in PBS, followed by blocking with 1% bovine serum albumin (BSA) in PBS. Actin microfilaments were stained with Alexa Fluor^®^ 488 phalloidin (green fluorescence) at a 1:100 dilution to visualize cell actin filaments. Cell nuclei were stained with DAPI (4′,6-diamidino-2-phenylindole) (blue fluorescence) at a 1:3000 dilution at room temperature. All reagents were obtained from Thermo Fisher Scientific (Villebon-sur-Yvette, France). The stained samples were maintained in 1% BSA in PBS before confocal imaging. Finally, images were captured using a TCS SP5 X confocal microscope (Leica, Wetzlar, Germany).

#### 2.2.4. Alizarin Red S Staining-Mineralization Potential

Alizarin red S staining was used to assess qualitatively and quantitatively matrix mineralization after 14 days of contact. Cell control and cellularized hydrogels were fixed using formaldehyde (3.7% in PBS, 30 min) and washed with deionized water. Then, 40 mM of alizarin red staining solution (pH 4.2) was added into the 24-well plates. The stained cells were incubated at room temperature for 40 min, then washed with deionized water 3 times and viewed under an optical microscope. For quantitative calcium analysis (semi-quantification) of mineralized matrix nodules generated from human gingival fibroblast cells, the samples were treated with 10% cetylpyridinium chloride solution (Sigma-Aldrich, Saint-Quentin-Fallavier, France) for 15 min at room temperature to dissolve and release the calcium-combined Alizarin red S into the solution. The optical density values were measured at 560 nm using a microplate reader (Infinite^®^ M200 PRO NanoQuant, Tecan, Chassieu, France), which indicated the relative quantity of mineralization nodules. The experiments were performed in triplicate.

#### 2.2.5. Statistical Analyses

Data were analyzed using statistical software SPSSTM (V21.0, IBM, Chicago, IL, USA) and found to be normally distributed. Non-parametric analysis and multiple comparison were achieved using one-way analysis of variance (ANOVA) with a repetition test followed by post hoc tests. A comparison was made between the functionalized hydrogel groups and the control hydrogel group. Results were reported as mean plus standard deviation (±SD), and statistical significance was accepted at *p* < 0.05.

## 3. Results

### 3.1. Physical Characterization

#### 3.1.1. Surface Chemical Composition by EDX

EDX mapping allows us to study the surface composition of hydrogels. Distinct compositions are observed between the control hydrogel and the functionalized hydrogels. SEM analysis was performed specifically to enable the examination of hydrogel surfaces using EDX. We acknowledge that the sample dehydration required for the SEM procedure could potentially damage the hydrogel structure.

**Figure 1:**
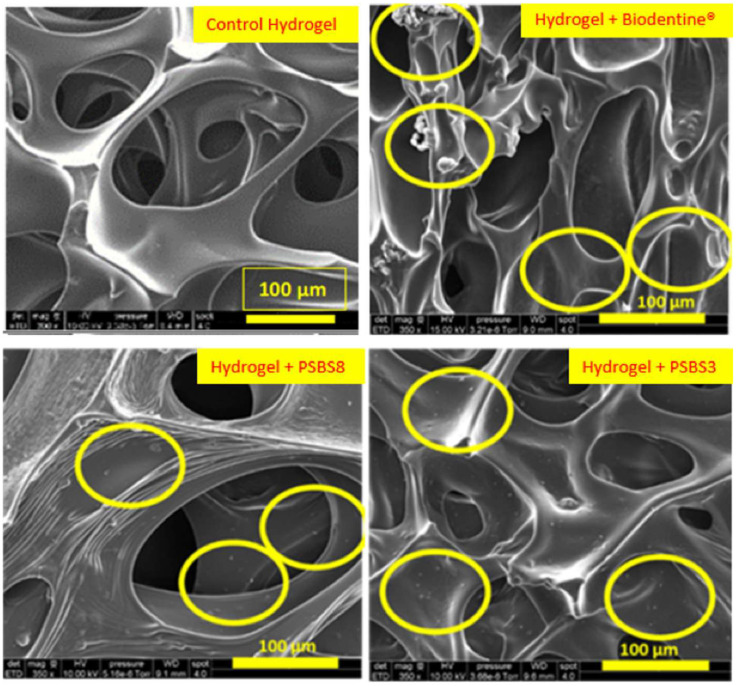
SEM image of functionalized hydrogels compared to the control hydrogel group (scale bars = 100 µm). Yellow circles indicate the presence of particles on the hydrogel surfaces.

This analysis reveals a porous structure in both non-functionalized and functionalized hydrogels, and it illustrates the integration of particles within the hydrogel network. The SEM images of the Biodentine^®^ hydrogel show more aggregates, attributable to its granulometry, which ranges between 500 µm and 1 mm. The experimental particles PSBS3 and PSBS8 have a particle size of less than 100 µm (Figure 1).

**Figure 2:**
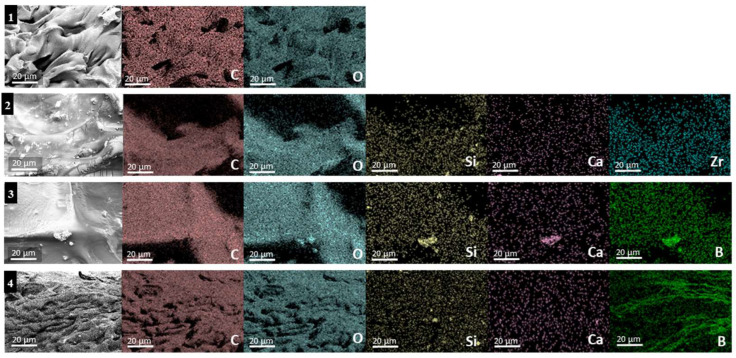
EDX mapping images of the tested functionalized gels: (**1**) Control gel, (**2**) Biodentine^®^ gel, (**3**) PSBS3 gel, and (**4**) PSBS8 gel, illustrating the morphology of various elements and their distribution profiles. The corresponding mapping areas are shown on the left side.

According to the EDX quantification results, PSBS8 contains more boron, followed by PSBS3, due to the composition of the experimental borosilicate. The absence of this element is noticeable on the surface of the Biodentine^®^ gel. The percentage of calcium is higher in the Biodentine^®^ gel, while the silica content is higher in the three functionalized gels compared to the control gel (Table 2 and Figure 2).

#### 3.1.2. pH Measurement and Calcium Ions Quantification

**Figure 3:**
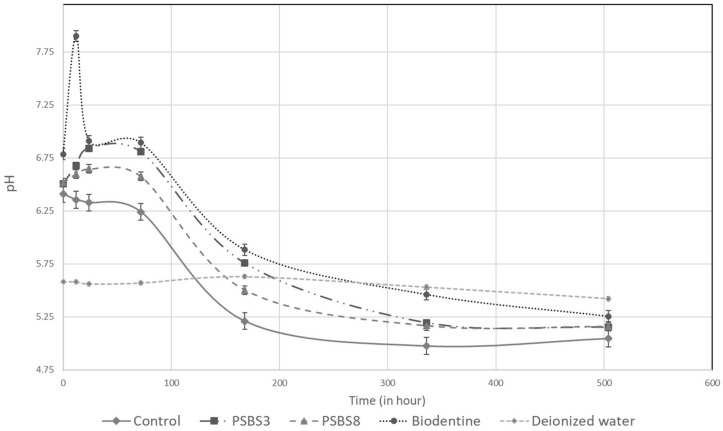
pH variations in control and functionalized hydrogels over time.

**Figure 4:**
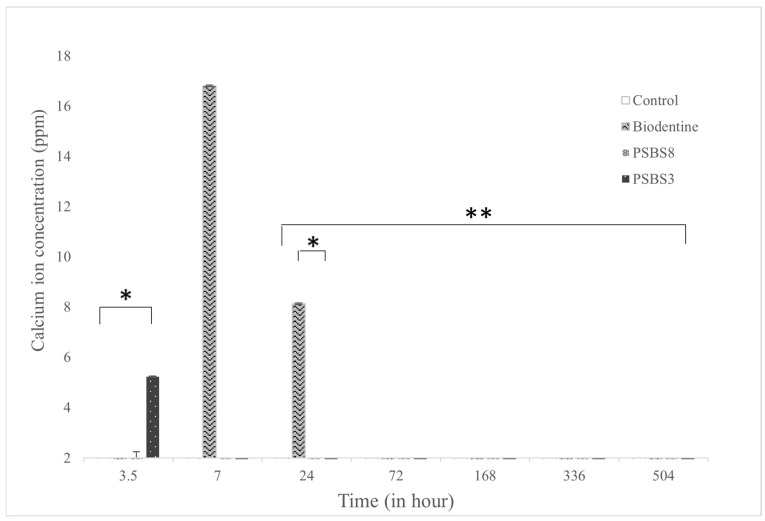
Time-dependent changes in calcium concentration. Data are mean ± SD of 3 independent experiments (n = 9). * *p* < 0.05 and ** *p* < 0.001 denote significant differences.

The results for pH measurement and calcium release are shown in Figure 3 and Figure 4, respectively. The pH values were more basic for the Biodentine^®^ gel, reaching a peak higher than that recorded for the two borosilicate gels, with the value increasing from 6.5 to 7.5 at 3 h of immersion. However, all pH values decreased starting from 7 h of immersion to the final time period (368 h). At all time points, the pH values of the control gel were lower than those of the functionalized gel, with stable values around 5.5. The amount of calcium released was significantly higher for PSBS3 at 3.5 h of immersion, followed by the PSBS8 gel, Biodentine^®^ gel, and finally the control gel (5.7 vs. 1.9, 1.7, and 0.2 ppm, respectively). After 7 h of immersion, the observed trend changed: Biodentine^®^ gel was the most effective with a value of 16.3 ppm, compared to a value around 2 ppm for the borosilicate gels. The evolution of calcium ions decreased after 72 h of immersion, reaching a value of 8 ppm for the Biodentine^®^ gel. The values for the borosilicate gels were almost stable, with a higher value for PSPS3

#### 3.1.3. Hydrogel’s Structure Using Micro-Computing Tomography (μCT) Analysis

**Figure 5:**
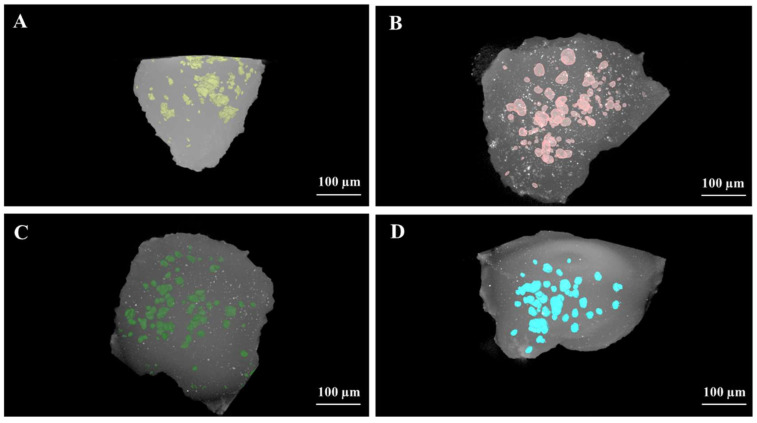
3D reconstructions of the tested hydrogels using ARIVIS 4.3 software: (**A**) control hydrogel, (**B**) Biodentine^®^ hydrogel, (**C**) PSBS8 hydrogel, and (**D**) PSBS3 hydrogel (scale bares = 100 µm).

The 3D renderings of the µCT volumes obtained from small specimens of the hydrogels are presented in Figure 5, with the corresponding void volume fraction and diameter data before and after 21 days of water aging provided in Table 3. The results indicate that the incorporation of particles (regardless of the particle types used) slightly influenced the hydrogel volume fraction in comparison to the control hydrogel, with average volume fractions of approximately 98.5% and 97.4% for the functionalized and control hydrogels, respectively. Regarding the average void size, an increase was observed for the two borosilicate functionalized hydrogels (122 µm for PSBS3 and 104 µm for PSBS8, compared to 87 µm for Biodentine^®^, and 79 µm for the control gel).

Aging in water for 21 days resulted in a reduction of more than 50% in pore volume fraction across all samples. Additionally, the two functionalized hydrogels exhibited a significant increase in void diameter post-aging, suggesting a transition to fewer but larger voids within the hydrogels.

#### 3.1.4. Thermogravimetric Analysis (TGA) and Differential Scanning Calorimetry Analysis (DSC)

**Figure 6:**
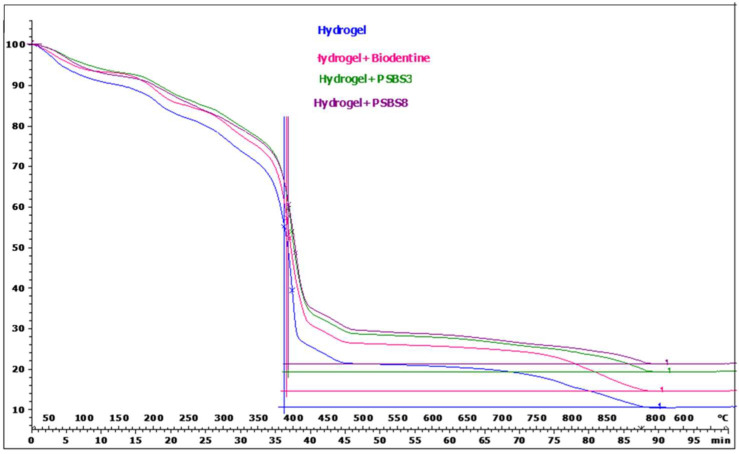
Representative TGA curve of the tested gels.

The TGA results are presented in Table 4, while the DSC results are shown in Table 5. Additionally, a representative TGA curve is illustrated in Figure 6. TGA experiments were conducted within a temperature range of 25 to 900 °C, employing a heating rate of 10 °C per minute, and utilizing nitrogen (N_2_) as both a protective and purging gas. Moreover, TGA was performed on the various hydrogel components to ascertain their respective melting points. The results indicate that the decomposition of the functionalized hydrogels follows that of the control hydrogel. The presence of BBG or Biodentine^®^ particles did not affect this behaviour. The resulting hydrogel thermograms depict changes in the trend characterized by the mass loss of a material as it undergoes degradation under specific temperature conditions (Table 4 and Figure 6). Regardless of the particles used for functionalization, an initial total mass loss of 9% occurred between 30 °C and 130 °C, corresponding to water evaporation. The highest total mass loss, 47%, was observed between 350 °C and 500 °C. The decarbonation observed between 820 °C and 810 °C may be attributed to the initial carbonate content in the hydrogel and the composition of Biodentine^®^.

**Figure 7:**
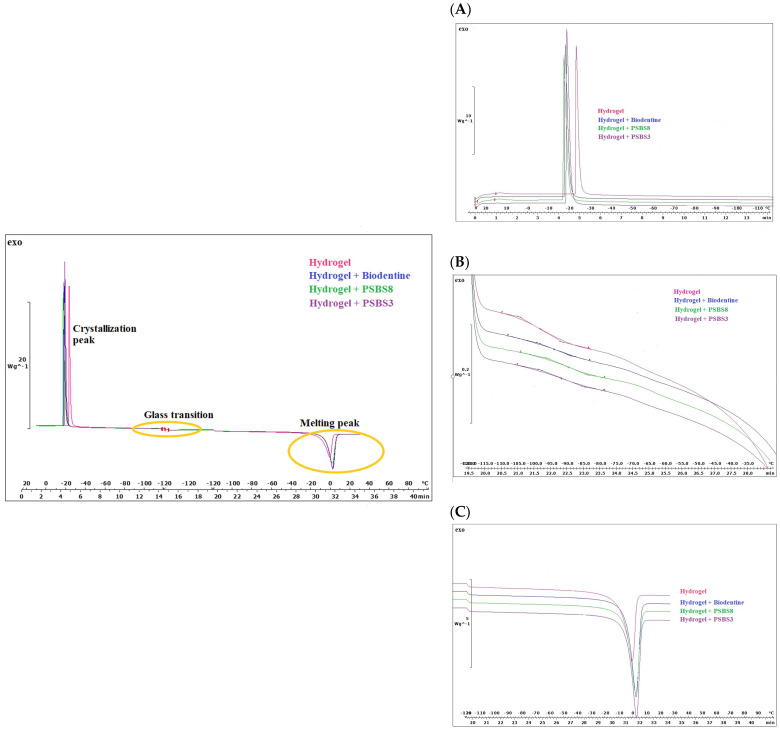
Representative DSC curve of the tested gels. (**A**) Peak temperature of crystallization, (**B**) glass transition temperature, and (**C**) peak temperature of melting.

DSC analysis was conducted over a temperature range of 25 °C to −120 °C, with a heating rate of 10 °C per minute. Water loss due to vaporization was considered negligible within this temperature range. The obtained DSC data exhibit distinct thermal phenomena, with slight shifts observed between the curves of each sample. Notably, functionalization of the hydrogels led to an increase in crystallization temperature (−18 °C for the control hydrogel compared to −12 °C for the functionalized hydrogels), consequently raising the melting temperature. Additionally, the glass transition temperature of PEG and DGL can be determined (Table 5 and Figure 7).

### 3.2. Results of the Cytocompatibility Assessment 

#### 3.2.1. Live/Dead^™^ Staining

**Figure 8:**
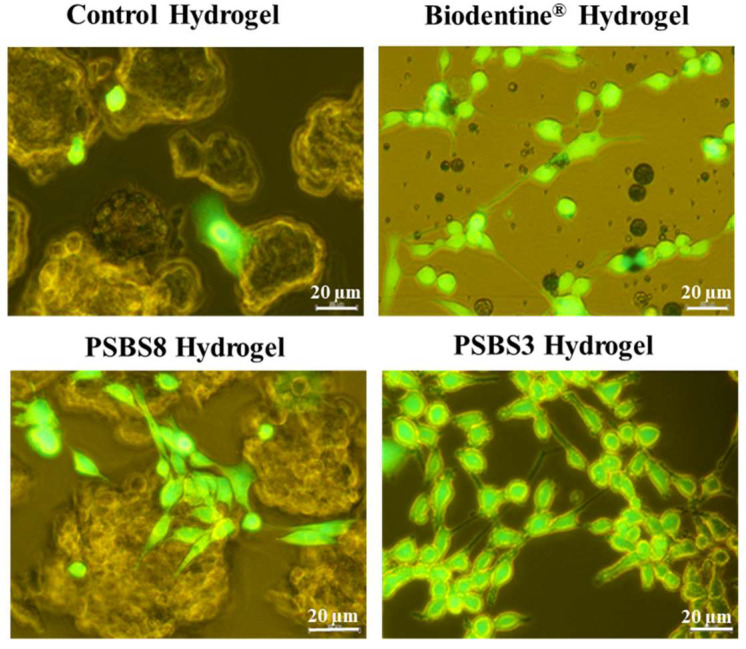
Assessment of HGF cytotoxicity using Live/Dead staining. Cells were observed under an epifluorescence microscope after 24 h of direct contact. Green areas indicate live cells, while red areas indicate damaged cells. No damaged (red) cells were observed. (Scale bars = 20 µm).

The Live/Dead staining images are presented in Figure 8. The images, acquired using epifluorescence microscopy, provide an assessment of cell proliferation via cell membrane integrity. Only green-stained cells were observed, indicating viable cells, while no red-stained cells were detected, suggesting the absence of damaged cells. These results indicate the absence of cytotoxicity in the presence of both functionalized and non-functionalized hydrogels.

#### 3.2.2. Cell Spreading and Hydrogel Colonization Using Confocal Imaging

**Figure 9:**
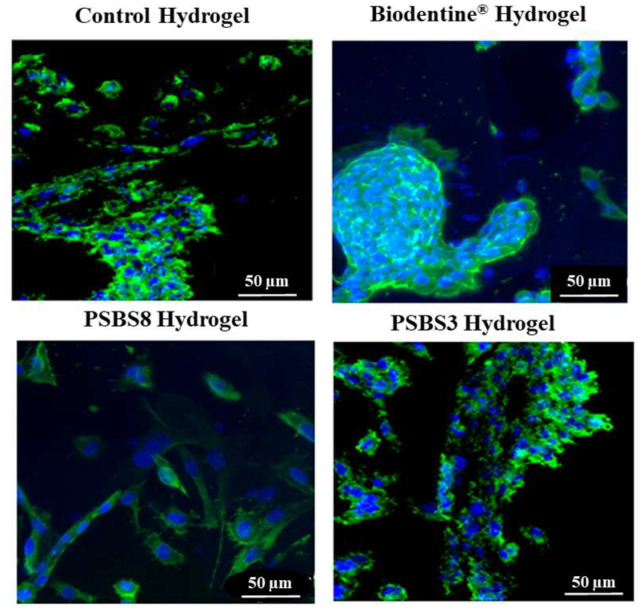
Spreading and colonization of hydrogels by HGF cells observed via confocal laser imaging after 24 h of direct contact with the various hydrogels (combination of DAPI-nucleus channels in Blue and Alexa Fluor^TM^ 488 Phalloidin. Cytoskeleton in green. Scale bars = 50 µm).

The HGF cells seeded within the tested gels are shown in Figure 9. In contact with all tested gels, cells showed an unaltered morphology and bright blue nuclei. Cells adhered to the gel walls, and their cytoskeletons followed the 3D hydrogel structure, indicating that cells colonized the porous gel structure and spread not only at the sample’s surface but also up to a depth of 200 µm. A higher cell density was observed in the presence of Biodentine^®^ and PSBS3 functionalization.

#### 3.2.3. Mineralization Ability by Alizarin Red Staining

**Figure 10:**
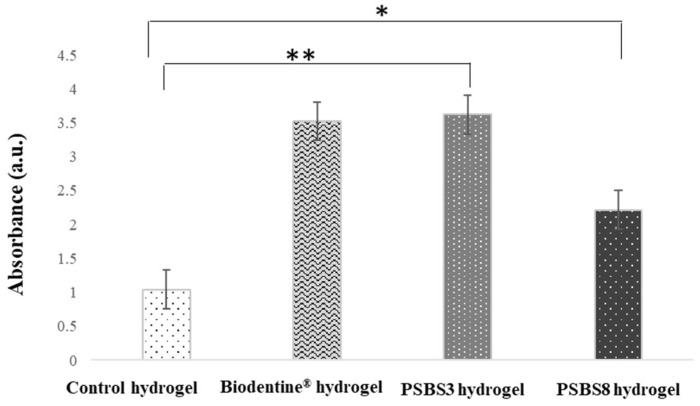
Quantification of calcium deposition by Alizarin red staining in HGF cells (B) as a function of contact time and hydrogel type. Data represent mean ± SD of 3 independent experiments (n = 6). * *p* < 0.05 and ** *p* < 0.001 indicate statistically significant values.

The mineralization potential of HGF cells in contact with the functionalized gels was determined using Alizarin red S staining after 7 and 14 days. After 14 days of incubation, a significant enhancement of extracellular calcium deposition was observed in particles compared to cells in contact with non-functionalized hydrogel. Mineralized nodules were noticeable under optical microscopy. Images demonstrated increased mineralization nodules when cells were interfaced with Biodentine^®^ and PSBS8 hydrogels compared to the control hydrogels (Figure 10). BBG functionalization significantly enhanced mineralization of HGF cells, albeit with less effectiveness compared to the Biodentine^®^ reference particles.

## 4. Discussion

The current challenge in dentistry is to develop minimally invasive therapies aimed at preserving the vitality of natural dental tissues [11]. Exploiting biocompatible, resorbable, and bioactive materials holds significant potential to effectively address this challenge. A recent review by Mirt et al. [28] highlighted the predominant bioactive glass formulations and their promising clinical applications in the biomedical field. The authors emphasized researchers’ efforts to develop composites based on bioactive glasses and polymers, particularly hydrogels, to enhance these materials’ properties. This interest has led to the creation of composite materials and injectable composites for minimally invasive administration. Bioactive hydrogels show potential in addressing various challenges by minimizing invasive surgeries, enhancing patient comfort, decreasing the risk of postoperative infections, and reducing treatment costs [28].

Hydrogels are three-dimensional, water-absorbing polymer networks that can be physically or chemically linked. The DGL/PEG hydrogels used in this study exhibit a high-water content, achieving a swelling ratio (Qs) of approximately 15 within 48 h, as reported by Griveau et al. [25]. These hydrogels act as barriers against bacterial infection and could form an environment suitable for tissue regeneration [29]. Another important feature of hydrogels is their ability to retain specific compounds while preserving their swollen state, even under varying pressures. Recent studies have highlighted the potential of hydrogels in dental applications. For instance, El-Fiqi et al. [30] demonstrated the enhanced regenerative capabilities of bioactive glass-incorporated hydrogels for dentin–pulp complex regeneration [30,31]. Similarly, recent studies have reviewed advanced hydrogels for periodontal tissue regeneration, highlighting the role of innovative crosslinking techniques in enhancing mechanical stability and biological activity [32,33,34]. Moreover, a study by Siddiqui et al. [35] explored the use of injectable hydrogels for pulp regeneration, showcasing their potential to support cell proliferation and differentiation. On the other hand, borosilicate bioactive glasses have recently demonstrated a high ion profile, good mineralization ability, and the capacity to stimulate dental pulp cell behaviour. These properties contribute significantly to enhancing the performance of hydrogels in dental tissue regeneration [9,11].

In the current study, porous hydrogels composed of polyethylene glycol (PEG) and poly(L-lysine) dendrimers (DGL) are synthesized via an effervescence reaction. This occurs when an acidic PEG solution interacts with a basic DGL solution, based on the protocol developed by Griveau et al. [25]. According to this study, the DGL/PEG hydrogels achieved a porosity of approximately 79%, with an average pore size of around 313 µm. Scanning electron microscopy (SEM) is employed to verify the porosity of these hydrogels, allowing for an evaluation of how functionalization affects hydrogel porosity.

The current EDX findings reveal the integration of particles within the hydrogel network, which supports the validation of the functionalized gels used in this study. SEM images of the Biodentine^®^ hydrogel show more significant aggregation, attributable to its granulometry ranging from 500 µm to 1 mm. In contrast, the experimental particles PSBS3 and PSBS8 exhibit a particle size of less than 100 µm. EDX is a widely used technique for analyzing elemental composition and distribution within materials. It has been employed in various studies to characterize the integration and dispersion of particles in hydrogel matrices [36].

The images acquired through micro-computed tomography (µCT) provide a detailed comparison of hydrogel morphology, particularly focusing on the influence of particles on the internal structure and the hydrogel volumetric fraction in each sample. µCT analysis indicates the internal presence of a small quantity of voids, which regardless of the particle type, were slightly reduced by functionalization of the hydrogels. This observation is surprisingly not representative of the extensively interconnected and highly porous structure obtained through the effervescent approach [37]. In addition, it has to be noticed that the pore morphology for the sample characterized through µCT is different from that characterized using SEM. The µCT images acquisition of the hydrogel in PBS and consequent lengthy exposure to x rays and heat might have altered the original structure of the hydrogels. Nonetheless, the effect of functionalization in reducing voids sizes is consistent with findings from a study by Unger et al. [36], which used µCT to investigate methacryloyl gelatin-based hydrogels functionalized with nanoliposomes.

The thermal behavior of the hydrogels is evaluated using thermogravimetric analysis (TGA) and differential scanning calorimetry (DSC). The objective is to compare the decomposition profiles and assess the influence of particles on their thermal behavior.

The TGA results indicate that the decomposition patterns of the functionalized hydrogels are consistent with those of the control hydrogel. However, DSC analysis reveals a slight positive shift in the crystallization and melting temperatures of the functionalized hydrogels. This observation suggests that the functionalization may affect the thermal properties of the hydrogels, particularly their glass transition.

In a related study by Ashames et al. [38], the incorporation of an active ingredient (cytarabine) into hydrogels resulted in changes in both crystallization and melting temperatures. This finding highlights the impact of additives on the thermal behavior of hydrogels, which differs from the effects observed in our study [38].

The average pH values and Ca^2^^+^ ion concentrations, measured from 3.5 h to 21 days, initially increase before eventually decreasing. The initial pH, around 5.5, corresponds to the pH of the deionized used water. Upon immersion of the functionalized hydrogels, the pH rises due to ion exchange reactions, with peak values observed between 1 and 3 days, as a result of the ionic exchange between the cationic network modifiers in the bio-glass particles and H_3_O^+^ in the aqueous medium. The most significant increase was observed for Biodentine^®^ due to the high hydrolysis potential of tricalcium silicate (Ca_3_SiO_5_). After this peak, the pH gradually declines with longer immersion times. This decrease in pH is likely due to the formation of hydroxyapatite (HA), which involves the uptake of OH^−^ ions from the surrounding medium [39]. The study of Prasads et al. [40] suggests that apatite formation becomes predominant after 3 days of immersion, which could explain this trend in pH evolution.

The calcium ion release, as measured by ICP-AES analysis, also shows a significant increase during the first 3 days of immersion, followed by a gradual decrease over time. Notably, there is a difference in the concentration of calcium released between the hydrogels functionalized with borosilicate glasses (PSBS3 and PSBS8) and the hydrogel functionalized with Biodentine^®^. Lizzi et al. [9] highlighted that the presence of different types of ions can influence the release of calcium ions. Biodentine^®^ has a higher calcium content, which accounts for the greater release of calcium observed. This study of Ca^2^^+^ release further supports the notion that the PEG- and DGL-based hydrogels can effectively release ions provided by the functionalizing particles, positioning them as potential vectors for dental tissues regeneration.

The results of the Live/Dead™ staining indicate that the addition of borosilicate particles to the porous did not affect its initial cytocompatibility. After one day, the cells were not damaged in direct contact with the hydrogels. The unfunctionalized PEG/DGL hydrogel was previously demonstrated to be cytocompatible [20]. Similarly, in the study conducted by Vagropoulou et al. [41] a chitosan/gelatin/nano-hydroxyapatite scaffold was synthesized. The Live/Dead™ test performed on this scaffold confirmed its non-cytotoxic nature, allowing for the human gingival cell proliferation, with over 95% of cells remaining viable.

The acquired confocal images demonstrated that the cells are well spread throughout the hydrogel. The nuclei, cytoskeleton, and pseudopodia of the cells are clearly visible. These images revealed a textured surface, indicating that the cells are actively colonizing the scaffolds. The functionalized hydrogels facilitate nutrient uptake and migration within their structure. This evidence confirms that the cells proliferate deeply and colonize the scaffold’s porous structure.

There are two primary methods for evaluating mineralization potential in vitro: the cellular approach, which involves stimulating the secretory capacity of cells to create calcium deposit nodules, and the acellular approach, which involves immersing the device in stimulated body fluid (SBF) buffer to form a hydroxyapatite (HA) deposit. The formation of an HA layer is generally considered an indication of cell-free bioactivity. The ability of bioactive glasses to form HA in body fluids is also utilized in toothpastes to treat dentinal hypersensitivity [42].

In the present study, the Alizarin red test performed on the hydrogels corresponds to the cellular approach, demonstrating a significant enhancement of the remineralization potential when primary gingival cells were interfaced with the functionalized hydrogels PSBS8 and Biodentine^®^. This quantitative assay confirms the bioactivity of the tested samples. Similarly, in the study of Mocquot et al. [43] the Alizarin red test on pulp cells showed that bioactive glass particles (sodium-free, synthesized by sol–gel) induce remineralization. This finding underlined that the particles incorporated onto the hydrogels preserved their bioactivity [43].

The PSBS3 and PSBS8 hydrogels are formulated from the same chemical species, but their compositions differ. Specifically, PSBS8 particles have a higher boron content and a lower calcium content compared to PSBS3 particles. In contrast, Biodentine^®^, used as a reference control, has a distinct chemical profile. These compositional variations could result in distinct properties among the hydrogels and could influence their performance in dental tissue applications. The observed differences in the behavior of PSBS3 and PSBS8 hydrogels stem primarily from variations in their chemical composition, specifically their boron and calcium contents. PSBS8, with its higher boron concentration and reduced calcium content compared to PSBS3, likely exhibits enhanced bioactivity and ion release characteristics due to boron’s role in promoting cellular responses and mineralization processes. Boron ions are known to support osteogenesis and angiogenesis, enhancing regenerative potential within dental tissues. In contrast, PSBS3, with its greater calcium content, supports quicker initial mineralization, which is essential for early tissue integration. However, the lower calcium levels in PSBS8 may slow initial mineralization but could simultaneously enhance long-term biocompatibility and bioactivity by preventing premature calcium crystallization, allowing for sustained ion release over time. These compositional differences collectively influence each hydrogel’s mineralization kinetics and cellular responses, with PSBS8 potentially offering prolonged regenerative benefits due to its boron profile, whereas PSBS3 supports a more immediate calcium-mediated response. Overall, the composition of PSBS8, with its boron-rich profile, may provide extended regenerative effects, while the higher calcium content in PSBS3 promotes a more immediate mineralization response. Further investigation is needed to fully understand these mechanisms and optimize their potential for a targeted clinical application.

Despite the limitations of this in vitro evaluation, such as the use of only one dental cell type, various parameters were assessed in accordance with the recommendations of the ISO 10993 standards to predict the in vivo biological behaviour of borosilicate-functionalized scaffolds as accurately as possible [44]. Further investigations, including the use of dental pulp cells and periodontal ligament cells, are needed to complete this evaluation and better mimic the other oral tissue behaviour. Moreover, the hydrogel crosslinking could be also adjusted in order to control the release of ions from the functionalized hydrogels over a longer period.

From a clinical perspective, borosilicate-functionalized hydrogels combine the bioactivity of borosilicate bioactive glasses with the flexible and porous structure of PEG/DGL hydrogels. This combination makes them a promising option for addressing the challenge of dental tissue regeneration within the framework of minimally invasive therapy development.

## 5. Conclusions

This study focuses on the development and assessment of a scaffold based on a PEG/DGL hydrogel functionalized with experimental borosilicate glass particles. Morphological and physicochemical characterizations of the functionalized hydrogels revealed that the addition of particles did not alter the hydrogel structure. Thermal analyses indicated that the functionalized hydrogels follow the decomposition profile of the unfunctionalized hydrogel under high temperature. The results obtained by ICP-AES and subsequent pH measurements confirmed that the hydrogel facilitates the release of calcium ions, which could promote acellular remineralization and contribute to the bioactivity of the experimental device. Cytocompatibility evaluations further indicated that the functionalized hydrogels exhibit no toxic effects when in contact with human gingival fibroblasts. Cellular remineralization tests demonstrated that, despite the brief release period of the ions, the functionalized hydrogels enhance remineralizing potential. Following additional investigations, the developed functionalized hydrogels demonstrate promising potential for application in clinical dental practice as a regenerative material.

## Figures and Tables

**Table 1:** Tested particle samples composition used to functionalize hydrogels.

	Composition (Mass%)
PSBS 3	25.0% B_2_O_3_; 0% Al_2_O_3_; 15.0% CaO; 45% SiO_2_; 15% K_2_O
PSBS 8	34.0% B_2_O_3_; 0% Al_2_O_3_; 6.0% CaO; 45% SiO_2_; 15% K_2_O
Biodentine^®^	80.1% Ca_3_ SiO_5_; 14.9% CaCO_3_; 5.0% ZrO_2_

**Table 2:** Chemical element rates at the tested hydrogel surfaces using EDX analysis.

Chemical Elements (%)	Control Hydrogel	Biodentine^®^ Hydrogel	PSBS8 Hydrogel	PSBS3 Hydrogel
C	61.27	81.56	21.04	29.68
O	38.66	7.95	45.83	54.79
Si	0.03	10.44	32.93	9.23
Ca	0.02	2.12	0.13	0.05
B	0.02	0	0.06	4.18

C: Carbon, O: oxygen, Si: silica, Ca: calcium, B: boron.

**Table 3:** Control and functionalized hydrogels Structures at Day 0 and day 21.

Samples	Control Gel		Biodentine^®^ Gel		PSBS8 Gel		PSBS3 Gel	
Immersion time period	D0	D21	D0	D21	D0	D21	D0	D21
Hydrogel volume fraction (%)	97.4	99.0	98.5	99.9	98.5	99.6	98.8	99.1
Void volume fraction (%)	2.6	1.0	1.5	0.1	1.5	0.4	1.2	0.5
Average void diameter (µm)	79	61	87	110	104	375	122	420

**Table 4:** Results of thermal analysis by TGA of the tested hydrogels.

	Onset Temperature of Mass Loss in °C		Mass Loss in %		
Decomposed Elements		Control Hydrogel	Hydrogel + Biodentine^®^	Hydrogel + PSBS8	Hydrogel + PSBS8
Removal of water from the hydrogel	30–130	9.548	6.857	7.808	6.946
Release of carbonyls or hydroxyls from lysine	130–260	8.558	8.433	6.760	6.369
Decomposition of lysine	260–350	8.743	8.754	4.373	6.230
PEG decomposition	350–500	47.712	45.954	45.170	45.858
Decomposition which may be linked to the species contained in the PBS	550–820	6.112	6.208	3.464	3.310
Decarbonation of K_2_CO_3_	820–810	4.660	5.458	2.962	4.148

**Table 5:** Thermograms analysis using DSC for the tested hydrogels.

	Temperature (°C)			
Observed Phenomenon	Control Hydrogel	Biodentine^®^ Hydrogel	PSBS8 Hydrogel	PSBS3 Hydrogel
Hydrogel crystallization temperature	−18.3	−12.7	−12.4	−12.3
Hydrogel melting temperature	−1.81	0.54	0.47	0.74
Hydrogel glass transition temperature	−98.1	−93.9	−89.5	−91.8

## Data Availability

The original contributions presented in the study are included in the article, further inquiries can be directed to the corresponding author.
